# EQ-5D utility, response and drug survival in rheumatoid arthritis patients on biologic monotherapy: A prospective observational study of patients registered in the south Swedish SSATG registry

**DOI:** 10.1371/journal.pone.0169946

**Published:** 2017-02-02

**Authors:** Tanja Schjødt Jørgensen, Carl Turesson, Meliha Kapetanovic, Martin Englund, Aleksandra Turkiewicz, Robin Christensen, Henning Bliddal, Pierre Geborek, Lars Erik Kristensen

**Affiliations:** 1 The Parker Institute, Copenhagen University Hospital, Bispebjerg and Frederiksberg, Copenhagen, Denmark; 2 Rheumatology, Department of Clinical Sciences, Malmö, Lund University, Malmö, Sweden; 3 Department of Clinical Sciences, Lund, Section of Rheumatology Lund University and Skåne University Hospital, Lund, Sweden; 4 Rheumatology, Department of Clinical Science, Lund University, Lund, Sweden; 5 Clinical Epidemiology Unit, Orthopaedics, Department of Clinical Sciences, Lund University, Lund, Sweden; Nippon Medical School Hospital, JAPAN

## Abstract

**Objectives:**

Biologic agents have dramatically changed treatment of rheumatoid arthritis (RA). To date only scarce head-to-head data exist especially when the biological therapies are given as monotherapy without concomitant disease modifying drugs (DMARDs). Thus the objective of the current study is to evaluate treatment response of all available biological therapies with special focus on utility (EQ-5D-3L) and drug survival of biologic DMARDs (bDMARDs) prescribed as monotherapy in RA patients in southern Sweden.

**Materials and methods:**

All RA patients registered in a regional database as initiating bDMARD as monotherapy, i.e. without concomitant conventional synthetic DMARDs (csDMARDs), from 1^st^ of January 2006 through 31^st^ of December 2012, were included. Patients were followed from initiation of the first dose of bDMARD monotherapy treatment until withdrawal from treatment, loss of follow-up or 31^st^ of December 2012. Descriptive statistics for utility (EQ-5D-3L), effectiveness, and drug survival of bDMARD monotherapy were calculated.

**Results:**

During the study period, a total of 554 patients were registered in SSATG as initiating bDMARD monotherapy. Most of the patients were women (81%), with a mean age of 57 years. The average disease duration was more than 12 years, and on average the patients had previously been treated with approximately four different csDMARDs. Fifty-five percent of the patients were initiating their first bDMARD, 26% their second, and 19% their third or more. At baseline the average EQ-5D-3L was 0.34. Most patients had moderate to high disease activity, with a mean DAS28 of 5.0, and were substantially disabled, with an average HAQ score of 1.4. At 6 months´ follow-up, the EQ-5D-3L in patients still on the biologic drug had increased by mean 0.23 (SD 0.4) with no differences between type of bDMARD (p = 0.49). The mean change in EQ-5D-3L ranged from 0.11 (rituximab and infliximab) to 0.42 (tocilizumab). Although the changes were numerically different, no distinct pattern favored any particular bDMARD for EQ-5D-3L (p = 0.49) or other clinical outcomes. Overall, DAS28 defined remission and low disease activity were achieved in 20% and 43% of patients, respectively. Drug survival rates were statistically significantly different between bDMARDs (p = 0.01), with the highest rates observed for rituximab, followed by etanercept. After failing first course of anti-TNF, patients switching to another mode of action had significantly higher drug survival than those switching to a second course of anti-TNF therapy (p = 0.02).

**Conclusions:**

Utility (EQ-5D-3L) increased after 6 months of all bDMARD treatments in monotherapy, indicating improvement of patients’ quality of life. After failure of anti-TNF treatment in monotherapy, switching to another mode of action may be associated with better drug survival than starting a second TNF-inhibitor.

## Introduction

Rheumatoid arthritis (RA) patients should be treated as early as possible with disease-modifying anti-rheumatic drugs (DMARDs) to improve the disease course [1]. Methotrexate (MTX) is considered the anchor drug in RA, both on the basis of its efficacy and safety as monotherapy, as well as its ability to increase the efficacy of biologic agents when used in combination [2–5].

It is estimated that between 10 and 30% of RA patients are MTX-intolerant and discontinuation is common in clinical practice [6]. Adverse effects from MTX include ulcerative stomatitis, leukopenia, liver toxicity, nausea and abdominal distress [7]. Thus, there are many reasons for discontinuation of MTX during biologic DMARD (bDMARD) therapy or initiating at least some of the bDMARDs as monotherapy. For those patients who are in need of treatment with a bDMARD and cannot tolerate MTX, bDMARD monotherapy may be a good option. Effectiveness and drug adherence of monotherapy with bDMARD has previously been described in a cohort study of patients with RA registered in the Danish DANBIO registry [8].

It is well known that RA has significant socioeconomic impact defined as health loss associated with diseases including both morbidity and mortality [9]. The Global Burden of Disease studies [10, 11] have recently addressed the challenges faced by the healthcare systems and, in this context, the cost-effectiveness of treating RA is highly impacted by modern therapies [12]. Thus, it can be claimed that rheumatologists need to show responsibility and consider economic implications when choosing between treatment options or modalities with comparable efficacy and safety. With growing numbers of biosimilar bDMARDs in the field of rheumatology, cost considerations by rheumatologists will become increasingly important [13]. In addition, stakeholders such as payers and administrators have to consider individual and societal implications (i.e. work disability and loss of productivity) of RA when making guidelines or treatment recommendations regarding the use and implementation of bDMARDs.

However, little is known about utility gain in clinical practice. Moreover, reports from independent cohorts in a similar clinical setting are lacking. The aim of this study was to investigate the frequency and type of bDMARDs used in monotherapy in a representative sample of patients with RA. Further, to evaluate and compare treatment response with special focus on utility (EQ-5D) and tolerability (drug adherence) among the different bDMARDs. For this purpose, we used the regional register held by the Southern Sweden Arthritis Treatment Group (SSATG), which covers more than 90% of rheumatology patients treated with bDMARDs [14] in southern Sweden setting, and can be considered representative of patients with RA who are treated in routine care.

## Materials and methods

Eligibility criteria, data summaries, and statistical analyses were based on a predefined protocol, which can be accessed through the first author. The outcomes were utility (EQ-5D-3L) gain, disease activity score in 28 joints (DAS28) remission, DAS28 defined low disease activity (LDA), the American College of Rheumatology response criteria 20%, 50%, 70% (ACR20, ACR50, ACR70) after 6 months of follow-up, and drug adherence.

### Study design, setting, and participants

RA patients who were registered in the SSATG database [14] as receiving initiation of treatment with any bDMARD without concomitant conventional synthetic disease-modifying anti-rheumatic drugs (csDMARD) therapy (i.e., monotherapy) in the period from 1^st^ of January 2006 through 31^st^ of December 2012 were included in the present study. There was no formal definition of the initiation date of bDMARD monotherapy. The initiation date was based on the decision of the treating physician, and most of the patients were exposed and intolerable to csDMARDs prior to the bDMARD monotherapy initiation. A minority (about 3%) of the patients had contra-indications for csDMARDs and was initiated directly on bDMARD monotherapy. The patients were continuously enrolled and were monitored in the SSATG register at baseline and during treatment. The patients were seen in the clinics at individual time points. Patients were followed from initiation of the first dose of bDMARD monotherapy treatment until withdrawal from treatment, loss of follow-up or 31^st^ of December 2012. Drug survival were retrieved from data recorded on withdrawals from treatment, which were routinely recorded by the treating physicians and classified as being due to adverse events, lack of response/inefficacy, or miscellaneous reasons such as pregnancies, patient decisions, poor compliance, and other unspecified causes. No formal criteria were required to terminate treatment, and the decision was based on the judgment of the treating physician.

The study sample included RA patients on monotherapy from bio-initiation and throughout the study period, whereas patients who became on monotherapy after cessation of concomitant csDMARD were excluded. A random sample of 50 patients from the SSATG database was validated by TSJ with respect to monotherapy timelines for date of onset (first dose bDMARD administered), dosage for the bDMARD monotherapy, and—in cases of withdrawal—the date of first missing dose. The validation showed complete data recording except for 1 patient (2%) with misrecorded termination date with monotherapy.

The regional ethics board of the University of Lund approved linkage of laboratory and clinical data used for this study (No. 379/2011). Informed consent was not required for the present study, as the study was part of a quality ensuring data recording process of routine clinical care, where all data handling was anonymous.

### Variables

At the time of inclusion in the SSATG register, the following core data were recorded: diagnosis, year of disease onset, previous csDMARD treatment, and previous bDMARD treatment. At treatment start (baseline) and at follow-up, utility gain (EQ-5D-3L based on British tariff to improve external validity) was calculated [15, 16] on those still on the biologic drug (EQ-5D-3L only exist on patients still on the drug). The EQ-5D is a five-dimensional health state classification. The five dimensions are mobility, self-care, usual activities, pain/discomfort and anxiety/depression. It is a preference-based measure that can be regarded as a continuous outcome scored on a -0.59 to 1.00 scale, with 1.00 indicating ‘full health’ and 0 representing dead [17]. The negative EQ-5D scores represent certain health states valued as worse than dead.

In addition, the following clinical data were registered: DAS28 remission, LDA, health assessment questionnaire score (HAQ), patient-scored visual analogue scales for pain (VASpain), general health (VASglobal), physician’s global assessment of disease activity scored on a visual analogue scale, 28 tender and swollen joint count, low disease activity, CDAI, SDAI and C-reactive protein (CRP). DAS28, CDAI, and ACR20, ACR50, and ACR70 responses compared to baseline were calculated at 6 months of follow-up. Patients were considered in remission with a DAS28 score less than 2.6, and LDA was defined as DAS28 less than 3.2 at follow-up.

### Statistical analyses

In the analysis the first monotherapy treatment for each patient during the study period was included (i.e. each patient was included once). Data were analyzed by Kruskal-Wallis test for between-group comparisons regarding continuous variables, whereas a Chi-square test was used for categorical variables. The Wilcoxon signed rank test was used to assess changes in EQ-5D from baseline to the 6 month follow-up. For the discrete treatment outcomes, both per-protocol and ITT-adjusted data (using the LUNDEX [18]) are given. The LUNDEX adjustment is an ITT methodology developed for the observational setting to account both for withdrawals from therapy and for missing response recordings at certain points of follow-up [18]. Drug survival data were illustrated by Kaplan Meier plots and further statistically analysed with log-rank statistics for comparing different treatments. Level of significance was chosen to be two-sided p<0.05.

## Results

**Fig 1:**
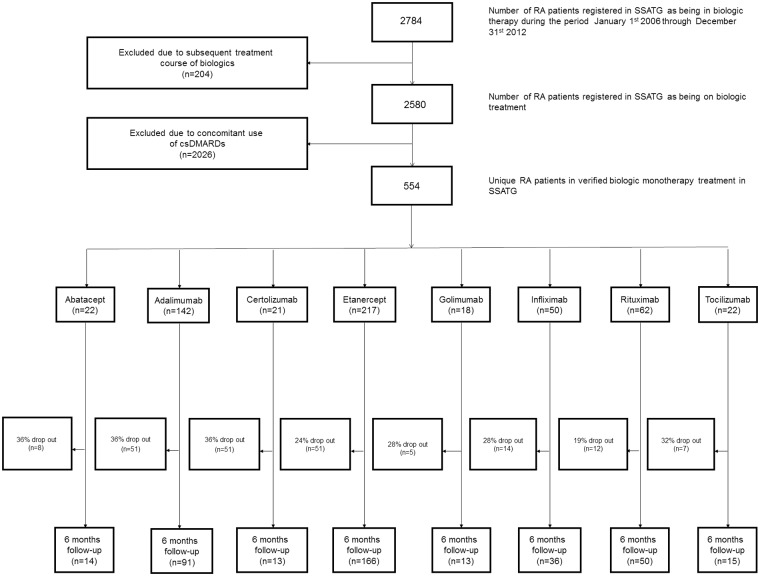
Study sample (flow diagram).

During the study period, a total of 554 patients were registered in SSATG as initiating bDMARD monotherapy, as seen in Fig 1.

At baseline most of the patients were women (81%), with a mean age of 57 years. The average disease duration was more than 12 years, and on average the patients had previously been treated with approximately four different csDMARDs. Fifty-five percent of the patients were initiating their first bDMARD, 26% their second, and 19% their third or more. As presented in Table 1, the average utility or preference-based measure of health (EQ-5D-3L) was 0.34. At the time of inclusion, most patients had moderate to high disease activity, with mean scores for DAS28 of 5.0, CDAI of 28.7, and SDAI of 31.1, and were substantially disabled, with an average HAQ score of 1.4 (Table 1).

**Table 1:** Baseline characteristics and disease activity of patients in the study population according to treatment.

Characteristics	Abatacept (n = 22)		Adalimumab (n = 142)		Certolizumab (n = 21)		Etanercept (n = 217)		Golimumab (n = 18)		Infliximab (n = 50)		Rituximab (n = 62)		Tocilizumab (n = 22)		Total (n = 554)	
		n		n		n		n		n		n		n		n		n
Female sex, no. (%)	19 (86%)	22	116 (82%)	142	19 (91%)	21	175 (81%)	217	14 (78%)	18	37 (74%)	50	48 (77%)	62	18 (82%)	22	446 (81%)	554
Age, years	53.0 (13.2)	21	57.2 (15.4)	140	52.0 (14.1)	21	56.0 (14.4)	216	59.5 (10.7)	18	62.0 (11.2)	47	58.6 (12.9)	62	57.5 (13.8)	22	57.0 (14.2)	547
Disease duration, year	11.2 (9.5)	21	11.7 (10.6)	140	10.3 (9.3)	21	12.6 (11.7)	216	11.5 (9.7)	18	18.5 (16.2)	47	13.6 (12.7)	62	10.1 (7.9)	22	12.7 (9.5)	547
No. of previous DMARDs	7.3 (3.4)	22	3.5 (2.1)	140	3.4 (2.5)	21	3.2 (2.2)	216	3.9 (3.3)	18	3.4 (2.0)	50	5.6 (3.3)	61	4.7 (2.6)	22	3.8 (2.6)*	550
No. of previous biologics	2.6 (1.4)	22	0.6 (0.7)	142	0.7 (1.0)	21	0.4 (0.8)	217	0.8 (1.0)	18	0.4 (0.8)	50	1.5 (1.3)	62	1.6 (1.5)	22	1 (NA)*	243
Biological treatment,																		
series no. (%)																		
1st serie	1 (5%)		73 (51%)		12 (34%)		152 (70%)		10 (56%)		35 (70%)		16 (26%)		6 (27%)		305 (55%)	
2nd serie	3 (14%)		55 (39%)		4 (23%)		49 (23%)		3 (17%)		9 (18%)		16 (26%)		6 (27%)		145 (26%)	
3rd+ serie	18 (81%)		14 (10%)		5 (43%)		16 (7%)		5 (27%)		6 (12%)		30 (48%)		10 (46%)		104 (19%)	
Missing	0 (%)	22	0 (0%)	142	0 (0%)	21	0 (0%)	217	0 (0%)	18	0 (0%)	50	0 (0%)	62	0 (0%)	22	0 (0%)	554
Swollen joint count: 0–28	6.0 (4.0;12.0)	21	7.0 (3.0;11.0)	120	6.0 (4.8;9.3)	18	7.0 (3.0;12.0)	195	9.0 (5.0;11.75)	16	7.0 (2.0;10.5)	45	5.0 (2.5;11.0)	61	5.5 (3.25;9.75)	20	7.0 (18.5;36.6)	496
Tender joint count: 0–28	12.0 (8.0;24.0)	21	8.0 (4.0;14.0)	119	7.0 (2.0;12.0)	18	7.0 (3.0;13.0)	195	7.5 (4.0;11.5)	16	7.0 (4.0;12.5)	45	7.0 (2.0;14.0)	61	6.0 (3.25;10.0)	20	8.0 (3.0;13.0)	495
C-reactive protein, mg/L	39.0 (40.7)	22	27.4 (35.6)	126	20.4 (31.8)	19	19.1 (23.4)	201	31.9 (43.1)	16	26.2 (32.1)	45	22.2 (26.6)	61	35.6 (38.6)	20	24.1 (30.7)	510
Erythrocyte sedimentation rate, mm/hour	41.2 (26.4)	22	36.3 (27.5)	122	36.3 (25.6)	16	27.9 (22.4)	196	35.9 (25.4)	12	36.3 (23.9)	41	32.0 (25.0)	61	44.4 (27.5)	19	32.9 (25.1)*	489
Patient global assessment, 0–100 mm VAS	67.4 (22.9)	22	63.5 (22.6)	126	59.3 (23.0)	19	62.9 (22.4)	200	60.5 (26.0)	16	64.0 (21.8)	43	60.0 (23.1)	57	69.1 (23.5)	19	63.0 (22.6)	502
Doctors global assessment (5-grade Likert scale)	2.6 (0.6)	21	2.2 (0.7)	120	2.3 (0.8)	18	2.3 (0.7)	194	2.3 (0.6)	16	2.1 (0.8)	45	2.2 (0.8)	61	2.3 (0.7)	20	2.2 (0.7)	495
Patient pain assessment, 0–100 mm VAS	70.4 (20.8)	22	66.0 (23.4)	126	62.1 (23.0)	19	62.8 (22.4)	201	58.8 (27.9)	16	65.3 (20.5)	43	56.4 (24.0)	57	67.6 (16.2)	19	63.4 (22.7)	503
EQ-5D	0.19 (0.3)	17	0.37 (0.4)	92	NA	0	0.32 (0.4)	158	0.59 (NA)	1	0.42 (0.4)	36	0.38 (0.3)	30	0.15 (0.3)	4	0.34 (0.4)	338
HAQ score, 0–3	1.7 (0.5)	22	1.4 (0.7)	124	1.4 (0.8)	19	1.3 (0.7)	200	1.3 (0.8)	16	1.5 (0.8)	42	1.5 (0.7)	57	1.4 (0.6)	17	1.4 (0.4)	497
CDAI	36.3 (15.1)	21	29.1 (12.5)	118	26.4 (10.7)	18	28.8 (13.7)	191	29.3 (11.2)	16	27.7 (11.9)	42	27.1 (13.6)	57	26.3 (10.5)	19	28.7 (13.1)	482
SDAI	39.8 (17.0)	21	31.6 (13.9)	116	28.6 (12.1)	18	30.8 (14.6)	189	32.5 (12.1)	16	29.9 (13.6)	41	29.4 (15.1)	57	30.0 (11.9)	19	31.1 (14.3)	477
DAS28	5.7 (1.3)	21	5.1 (1.3)	116	4.7 (1.2)	18	4.9 (1.3)	190	5.1 (1.0)	16	5.0 (1.3)	41	4.8 (1.4)	57	5.1 (1.0)	19	5.0 (1.3)	478

Data are given as mean and standard deviation (SD) or median and interquartile [Q1;Q3] unless otherwise indicated.

*Statistical significant difference between the different biologic agents (p<0.05)

Of the 554 patients in monotherapy, adalimumab (25.6%), etanercept (39.2%), rituximab (11.2%), and infliximab (9.0%) were the 4 most prevalent bDMARDs administrated (Fig 1).

### Utility measure of health

At 6 months´ follow-up, the EQ-5D-3L in patients still on the biologic drug increased by mean 0.23 (SD 0.4) (p<0.001), indicating improvement of patients’ quality of life. The mean change in EQ-5D-3L ranged from 0.11 (rituximab and infliximab) to 0.42 (tocilizumab) (Table 2). Although the increases in EQ-5D-3L were numerically different, there was no statistically significant difference across the various bDMARDs (Table 2).). There were no major differences in sex (83% vs 78% females; p = 0.24), age (mean: 59.2 years vs 59.3 years; p = 0.37), disease duration (mean: 15.6 years vs 14.4 years; p = 0.55), and DAS28 (mean: 5.1 vs 4.9; p = 0.16) between patients reporting EQ-5D data and those with missing data.

**Table 2:** Utility gain and effectiveness of different biologics at 6 months follow-up.

Characteristics	Abatacept (n = 14)	Adalimumab (n = 91)	Certolizumab (n = 13)	Etanercept (n = 166)	Golimumab (n = 13)	Infliximab (n = 36)	Rituximab (n = 50)	Tocilizumab (n = 15)	Total (n = 398)	P-*value*
**ΔEQ-5D**										
**N**	6	36	0	91	0	21	15	2	171*	
	0.16 (0.4)	0.13 (0.4)	NA	0.31 (0.3)	NA	0.11 (0.5)	0.11 (0.3)	0.42 (0.5)	0.23 (0.4)	0.493
**DAS28 remission**										
**N**	11	83	10	148	13	35	38	15	353	
Remission, no (%)	0 (0%)	22 (27%)	2 (20%)	34 (23%)	3 (23%)	1 (3%)	7 (18%)	3 (20%)	72 (20%´)	
LUNDEX corrected (%)	0%	18%	13%	18%	13%	2%	15%	12%	13%	0.098
**DAS28**										
N	11	83	10	148	13	35	38	15	353	
	4.5 (1.7)	3.7 (1.5)	3.5 (1,4)	3.5 (1.3)	3.7 (1.3)	4.2 (1.1)	3.7 (1.6)	3.3 (1.1)	3.7 (1.4)	0.052
**ΔDAS28**										
**N**	11	73	9	139	13	31	35	13	324	
	-1.2 (0.9)	-1.4 (1.6)	-1.4 (1,7)	-1.5 (1.4)	-1.4 (1.2)	-0.9 (1.5)	-1.3 (1.2)	-1.9 (1.2)	-1.4 (1.4)	0.424
**DAS28 low disease activity (LDA)**										
**N**	11	83	10	148	13	35	38	15	353	
LDA, no (%)	3 (27%)	37 (45%)	5 (50%)	65 (44%)	4 (31%)	12 (34%)	15 (40%)	10 (67%)	151 (43%)	
LUNDEX corrected (%)	17%	29%	32%	34%	18%	22%	33%	39%	28%	0.428
**CDAI**										
**N**	11	82	10	147	13	36	38	15	352	
	24.0 (18.7)	18.1 (12.7)	13.4 (7.9)	15.1 (10.7)	15.3 (9.4)	17.6 (8.7)	17.7 (14.8)	14.5 (6.5)	16.6 (11.6)	0.152
**ΔCDAI**										
**N**	11	74	9	139	13	32	35	13	326	
	-11.6 (8.8)	-12.8 (15.0)	-16.0 (12.5)	-14.8 (14.1)	-15.0 (14.8)	-10.8 (13.0)	-12.6 (13.1)	-13.1 (10.3)	-13.6 (13.8)	0.533
**HAQ improvement**										
**N**	12	72	9	129	10	29	33	10	304	
Improvement > 0.3, no (%)	5 (42%)	21 (29%)	4 (44%)	45 (35%)	3 (30%)	9 (31%)	6 (18%)	2 (20%)	95 (31%)	0.599
LUNDEX corrected (%)	27%	19%	28%	27%	17%	20%	15%	12%	20%	
**ACR response**										
**N**	9	51	4	88	10	21	24	11	218	
ACR20, no (%)	6 (67%)	44 (86%)	3 (75%)	76 (86%)	9 (90%)	14 (67%)	18 (75%)	9 (82%)	180 (83%)	0.338
LUNDEX corrected (%)	43%	56%	47%	67%	52%	43%	62%	48%	54%	
**N**	7	43	3	69	5	19	20	9	175	
ACR50, no (%)	2 (29%)	25 (58%)	2 (67%)	45 (65%)	3 (60%)	9 (47%)	9 (45%)	2 (22%)	97 (55%)	0.165
LUNDEX corrected (%)	19%	38%	42%	51%	35%	30%	37%	13%	36%	
**N**	5	32	3	56	3	17	16	9	141	
ACR70, no (%)	0 (0%)	10 (31%)	1 (33%)	14 (25%)	0 (0%)	2 (12%)	1 (6%)	2 (22%)	30 (21%)	0.345
LUNDEX corrected (%)	0%	20%	21%	20%	0%	8%	5%	13%	14%	

N is the number of patients with complete recording of the particular outcome. ΔEQ5D, DAS28, ΔDAS28 CDAI and ΔCDAI are given as mean and standard deviation (SD). DAS28 remission, LDA, HAQ and ACR responses are given as no. and percent (%).

*The overall change from 0 to 6 months in EQ-5D was highly significant; p<0.001.

### Treatment responses

At 6 months´ follow-up, the crude DAS28 remission rates ranged from 0% (abatacept) to 27% (adalimumab); LDA ranged from 27% (abatacept) to 67% (tocilizumab); ACR20 response rate from 67% (abatacept and infliximab) to 90% (golimumab); ACR50 response rate from 22% (tocilizumab) to 67% (certolizumab); and ACR70 response rate from 0% (abatacept and golimumab) to 33% (certolizumab). Improvements of more than 0.3 in HAQ ranged from 18% (rituximab) to 44% (certolizumab) (Table 2). Although the rates were numerically different, these differences were not statistically significant (Table 2).

Overall, the crude mean change in DAS28 after 6 months was -1.4 and for CDAI -13.6 (Table 2). For DAS28 remission, the crude and LUNDEX corrected rates were 20% and 13%, respectively, and for LDA the crude and LUNDEX corrected rates were 43% and 28%, respectively. The crude and LUNDEX corrected response rates for ACR20/50/70 were 83%/55%/21% and 54%/36%/14%, respectively (Table 2).

### Drug survival, stratified by bDMARD

**Fig 2:**
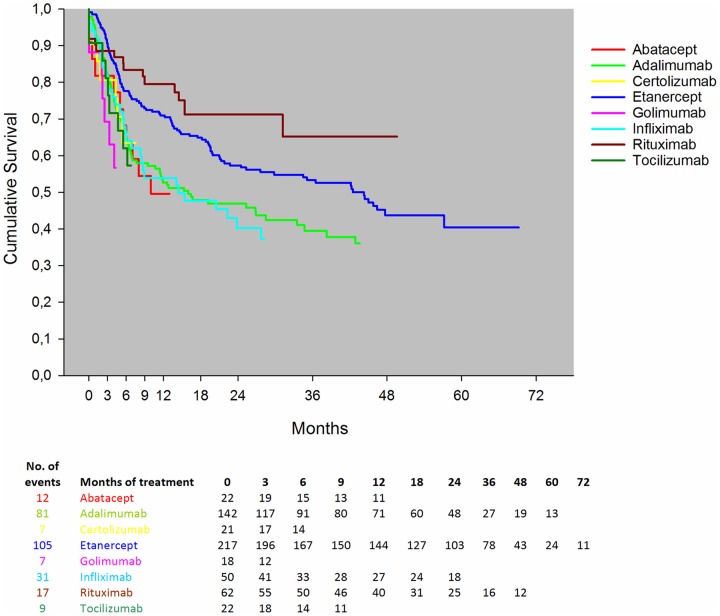
Drug survival, stratified by drug. The graph for the Kaplan-Meier estimated survival has been censored when the number at risk was below 10. The number of patients on a drug at different time points is listed below the figure.

A total of 156 patients withdrew from treatment during the 6 months´ follow-up (Fig 1). The overall drug survival rates were significantly different between the bDMARDs (p = 0.01). Rituximab showed statistically significantly better drug survival than etanercept (p = 0.05), whereas etanercept showed statistically significantly better drug survival rates compared to both infliximab and adalimumab (Fig 2). The estimated overall drug survival was approximately 65% after 6 months, declining to approximately 55% after 2 years, with the exception of abatacept, certolizumab, golimumab and tocilizumab having low number of patients and a short follow-up time (Fig 2).

### Drug survival in bDMARD switchers, anti-TNF vs. other modes of action

**Fig 3:**
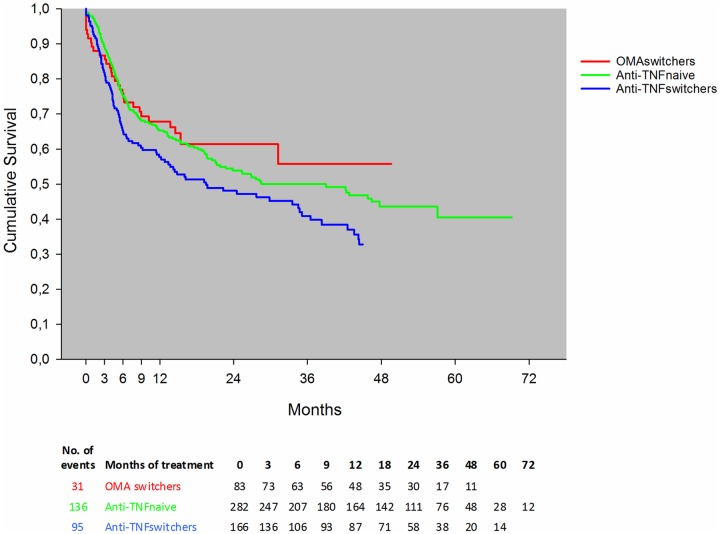
Drug survival, stratified by anti-TNF naive, anti-TNF switchers and other modes of action switchers (OMA switchers) in biologic monotherapy. The graph for the Kaplan-Meier estimated survival has been censored when the number at risk was below 10. The number of patients on a drug at different time points is listed below the figure.

**Fig 4:**
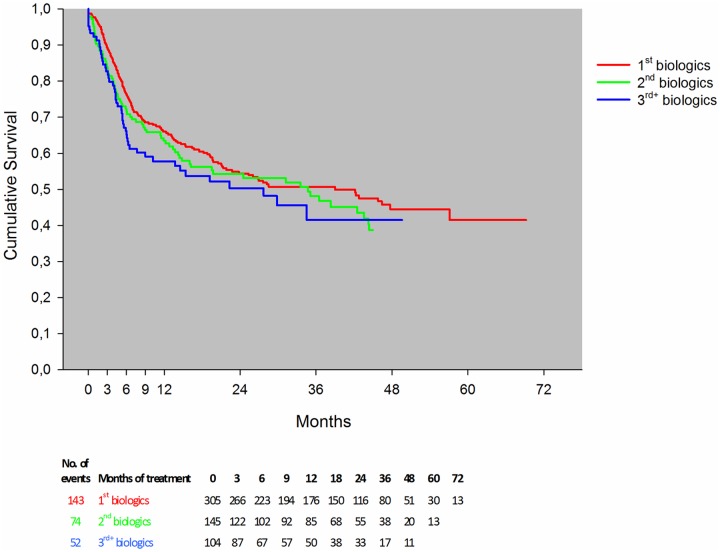
Drug survival, stratified by 1^st^, 2^nd^ and 3^rd^ line biologic monotherapy. The graph for the Kaplan-Meier estimated survival has been censored when the number at risk was below 10. The number of patients on a drug at different time points is listed below the figure.

The estimated drug survival rates were statistically significantly different when comparing anti-TNF switchers to other modes of action switchers (OMA switchers) (p = 0.02; Fig 3). There was no statistically significant difference between anti-TNF naïve patients and OMA switchers (p = 0.30) (Fig 3). The observed drug survival rates were similar irrespective of the number of biologic treatments the patients had received previously (p = 0.41; Fig 4).

There were no statistically significant differences between anti-TNF naïve patients, anti-TNF switchers, and OMA switchers regarding change in EQ-5D-3L, DAS28, HAQ, CDAI, and the crude and LUNDEX corrected ACR response rates (Table 3) at 6 months follow-up.

**Table 3:** Utility gain and effectiveness of anti-TNF naïve, anti-TNF switchers and other modes of action (OMA) bDMARDs 6 months follow-up.

	Anti-TNF naïve (n = 269)	Anti-TNF switchers (n = 160)	Other modes of action (OMA) (n = 83)	P-*value*
**ΔEQ-5D**				
**N**	58	31	9	
	0.28 (0.3)	0.25 (0.4)	0.14 (0.3)	0.379
**ΔDAS28**				
**N**	93	41	27	
	-1.6 (1.4)	-1.6 (1.3)	-1.7 (1,1)	0.897
**ΔCDAI**				
**N**	94	45	27	
	-15.4 (13.2)	-16.4 (11.7)	-15.6 (11.7)	0.680
**ΔHAQ**				
**N**	93	44	25	
	-0.3 (0.6)	-0.3 (0.5)	-0.1 (0.5)	0.100
**ACR response**				
**N**	189	82	51	
ACR20, no (%)	105 (56%)	37 (45%)	25 (49%)	0.261
LUNDEX corrected (%)	41%	29%	36%	
**N**	189	82	51	
ACR50, no (%)	61 (32%)	20 (24%)	11 (22%)	0.202
LUNDEX corrected (%)	13%	15%	16%	
**N**	189	82	51	
ACR70, no (%)	61 (32%)	20 (24%)	11 (22%)	0.202
LUNDEX corrected (%)	13%	15%	16%	

N is the number of patients with complete recording of the particular outcome. ΔEQ5D, ΔDAS28, ΔCDAI, and ΔHAQ are given as mean and standard deviation (SD). ACR responses are given as no. and percent (%).

## Discussion

In the present study, based on the data from the south Swedish SSATG registry, we found that the most common drugs used as biologic monotherapy were adalimumab, etanercept, and rituximab. One of our main findings was that in RA patients receiving bDMARD monotherapy, utility or preference-based health (EQ-5D-3L) on average nearly doubled after 6 months of treatment with a bDMARD, indicating improvement of patients’ quality of health [19, 20]. Whether the absolute changes are clinically important remains to be validated in the RA population. Although the changes were numerically different, no distinct pattern favored any particular bDMARD regarding change in health utility (EQ-5D-3L), remission and ACR response criteria. Furthermore, the estimated survival rates were generally lower than seen in combination therapy [21]. In addition, the survival rates seemed more favorable for rituximab than for etanercept, whereas, etanercept showed significantly better drug survival rates compared to both infliximab and adalimumab when given in monotherapy. On the other hand, LUNDEX corrected treatment response rates and proportions achieving DAS28 defined remission or low disease activity were not substantially different in patients treated with rituximab, etanercept and adalimumab.

The observed increase in health utility is in line with the utility gain found in an observational study by Gülfe et al. [22] and in RCTs of biologic therapy in patients with RA receiving concomitant csDMARDs [23, 24]. Comparison of change in utility score between bDMARD monotherapy group and combination therapy group would be a clinically important issue to investigate. However, with regard to background csDMARD usage this imposes an immense confounding by indication, as these different patient groups are very heterogeneous in the clinical setting [21]. Furthermore, this was not the scope of the current study, and must be addressed in future studies.

EQ-5D was chosen as utility outcome because of its simplicity, patient acceptability and well-established utilities. This adds important knowledge from a societal or payers perspective. This being said, EQ-5D is well suited for measuring health-related quality of life, including dimensions such as pain, mobility, self-care and usual activities, all of which are important in inflammatory joint diseases. However, EQ-5D entails several subjective judgments made by the patients, wherefore it has major limitations for clinical monitoring and has to be complemented with more objective measures before making decisions regarding the start or change of bDMARD treatment.

Significantly better drug survival rates for etanercept compared to both infliximab and adalimumab have previously been shown in independent RA cohorts treated with csDMARDs combination therapy [21, 25, 26], but it is to our knowledge the first time that this is demonstrated in a unique monotherapy cohort. The finding that rituximab should be even less likely to be discontinued is novel and could in part be due to methodologic issues. For example, the pattern of administration with long inter-infusion times for rituximab probably contributes to artificially improved drug survival in this analysis. As the study included more than four years of follow-up, these patterns may also reflect true differences between bDMARDs in terms of long term efficacy and safety. Of note, rituximab is often administered at a later state in the treatment algorithm, wherefore limitation in the remaining treatment options might inflate the drug survival rate in favor of rituximab. Future studies are needed to confirm our finding and explore the underlying mechanisms.

Surprisingly, we found that when switching to OMA after anti-TNF failure the drug adherence was as good as when treated with an anti-TNF as 1st course. However, switching to a 2^nd^ anti-TNF showed significantly inferior drug survival rates compared to bDMARDs with other modes of action. Recently, an observational study comparing rituximab to anti-TNF in combination therapy also showed superiority when switching to OMA compared to another anti-TNF [27]. Thus our results suggest that bDMARDs with other modes of action could be considered as the first choice for patients not tolerating csDMARDs and who have had an inadequate response to their first anti-TNF agent. It should be noted, that the favorable effect of OMA is primarily driven by rituximab.

The clinical remission and response rates in the present study are comparable with results from a study in the Danish DANBIO registry [8] showing somewhat lower overall remission and response rates than in previously published studies in bDMARD therapy combined with MTX [21, 25, 28–33].

The overall retention rate after 6 months was approximately 65%, declining to approximately 55% after 2 years, which is lower than the result observed in the DANBIO registry [8] and generally lower than previously published studies on combination therapy [21, 26, 29–32]. The reason for the discrepancies in drug survival between SSATG and DANBIO might be due to various reasons such as differences in the data capturing, regional prescription policies, and the fact that the DANBIO report also included patients starting out in combination therapy, but who withdrew from csDMARD during the course of the particular bDMARD.

In line with the monotherapy study from DANBIO [8], the survival rates found in the present study were independent of previous treatments. Patients who were in their 2^nd^ or 3^rd^ line of bDMARD as monotherapy adhered to the treatment as long as those who were on their first biologic agent on monotherapy. This finding is in contrast with data from other registries on RA patients in combination therapy [34, 35], showing that drug survival in switchers was lower than in anti-TNF naïves [34, 36] and an increased risk of withdrawing from the second treatment for the same reason as the first, regardless of whether it was due to inefficacy or adverse events [35]. Other possibilities might be the pooling of anti-TNF bDMARDs and bDMARDs with other modes of action in the present analysis which may have influenced the drug survival in either direction for switchers, or that adherence to monotherapy depends more on other factors (e.g., patients’ age and comorbidities).

### Limitations and strengths

The non-randomized observational design of this study has limitations such as possible bias regarding patient selection, assignment of treatment, baseline disparities between groups and missing data. The patient background features and conditions were different, so one could argue whether direct comparisons for outcomes among biologic agents is appropriate. Confounding by indication is an issue in the current study, being the inherent problem with all observational studies. Thus, we have faded the role of direct comparison by grouping the different agents in larger groups (anti- TNF’s and OMA) in the main analysis performed. Moreover, regression-analysis was performed to compensate for confounding, however, residual confounding always remains a problem in the observational study setting.

The Swedish national bDMARD registry [37] demonstrated that patients with many comorbidities might have been channeled to non-TNF bDMARDs hampering the effectiveness of these drugs. Moreover, TNF inhibition has previously been first line treatment in RA, wherefore non-anti-TNF agents have been used more extensively as 2^nd^ or 3^rd^ line treatment, potentially deflating the response to these bDMARDs. This being said, summary disease characteristics in this study were similar across different treatments, suggesting a limited potential role of such confounding. Furthermore, decisions to start or stop therapy as well as concomitant DMARDs were based solely on clinical practice and experience of treating physicians, with national guidelines as a support.

Major strengths of the present study include access to prospectively recorded, routinely collected registry data on biologic treatment. Observational data like ours are more generally applicable as a reference for health economic modeling than randomized controlled trial data, which are derived from highly selected patients. Observational studies remain important contributors of information from daily clinical practice.

## Conclusions

This observational study showed that utility (EQ-5D-3L) significantly increased after 6 months of treatment with a bDMARD when applied in monotherapy, indicating improvement of patients’ quality of life. Although there were numerical differences between bDMARDs in terms of utility gain and clinical response, no distinct pattern favored any particular bDMARD when used as monotherapy for RA. Survival rates were more favorable for rituximab than for etanercept, whereas etanercept showed significantly better drug survival rates compared to both infliximab and adalimumab. Furthermore, after anti-TNF failure, switching to a 2^nd^ anti-TNF drug showed significantly inferior drug survival rates compared starting one of the bDMARDs with other modes of action.
